# Effects of a National Preventive Intervention Against Potential COVID-19–Related Gambling Problems in Online Gamblers: Self-Report Survey Study

**DOI:** 10.2196/33066

**Published:** 2022-03-09

**Authors:** Anders Håkansson, Andreas Sundvall, Axel Lyckberg

**Affiliations:** 1 Department of Clinical Sciences Lund, Psychiatry Faculty of Medicine Lund University Lund Sweden; 2 AB Svenska Spel Visby Sweden

**Keywords:** gambling disorder, problem gambling, COVID-19, harm reduction, behavioral addiction

## Abstract

**Background:**

The COVID-19 pandemic has been suspected to increase gambling problems in the population. Several governments introduced COVID-19–specific interventions early with the aim to prevent gambling problems, but their effects have not been evaluated.

**Objective:**

This study aimed to evaluate a Swedish COVID-19–related temporary legislation imposing an automated weekly deposit limit for online casino gambling.

**Methods:**

The study was an anonymous survey sent by a state-owned gambling operator to online gamblers (N=619), among whom 54.0% (n=334) were moderate-risk/problem gamblers who reached the weekly limit on online gambling during the summer of 2020.

**Results:**

Overall, 60.1% (372/619) were aware of having been limited by the COVID-19–related deposit limit, and a minority (145/619, 23.4%) perceived the intervention as fairly bad or very bad. Among those aware of the intervention, 38.7% (144/372) believed the intervention decreased their overall gambling, whereas 7.8% (29/372) believed it rather increased it. However, 82.5% (307/372) reported having gambled at more than one operator after the limit, and the most common gambling type reported to have increased at another operator was online casino (42% among moderate-risk/problem gamblers and 19% among others; *P*<.001). An increase in gambling following the intervention was associated with being a moderate-risk/problem gambler and having negative attitudes toward the intervention.

**Conclusions:**

The weekly deposit limit had relatively high acceptability, but the study highlights the limitations of a single-operator deposit limit, given the high number of gamblers also reporting gambling at other operators and the lower effect in clients with gambling problems.

## Introduction

The global spread of SARS-CoV-2, causing the COVID-19 pandemic, is resulting in a number of mental health consequences in the population [1] caused by either the direct effects of the disease or the restrictions imposed on society and the following behavioral changes. One of the public health hazards suggested to be caused by the pandemic is gambling. Increased gambling behavior, at least in subgroups in the population, was feared in the early phases of the pandemic [2]. A priori, problem gambling, including in the group of individuals who meet the criteria of having a gambling disorder, constitutes a well-known hazard to health and a risk of psychosocial problems [3]. An overview of the research hitherto conducted in the area of gambling behaviors during COVID-19 recently demonstrated the different findings of the studies conducted so far, with several studies demonstrating that gambling practices rather decreased or did not increase, but some reporting an increase among subgroups, including individuals with a higher degree of gambling problems [4].

Thus, altogether, there are hitherto no convincing findings of generally increased gambling due to COVID-19, but there are indications that the potential effects are very unevenly distributed in the population. Sweden in one of the settings where a number of studies have assessed objective gambling activity measures, or self-reported survey data, during COVID-19. Objective measures of gambling activity have shown that gambling at online commercial gambling operators did not increase during the very first phase of the pandemic, when sports events generally were cancelled [5,6]. However, the financial activity of gambling operators demonstrated some likely migration of gambling behavior away from traditional sports betting during the early period of the sports lockdown [7]. In line with the overall picture of a potential gambling increase in subgroups of the population, an early survey study [8] and its similar follow-up [9] demonstrated that 4% and 6%, respectively, stated a self-reported increase in gambling after the onset of the pandemic, with a considerable overrepresentation of individuals with moderate-risk or problem gambling in this group. As an additional data source in this context, treatment uptake at a regional health care facility for gambling disorder patients was not statistically changed by the COVID-19 pandemic [10].

A number of countries took early action with legislation aiming to prevent problem gambling in response to the pandemic. This included gambling bans, bans against gambling advertising, or limits on gambling deposits, for example, in the United Kingdom, Spain, and Latvia [11-13]. A COVID-19–related legislation, effective from July 2, 2020, was also introduced in Sweden, as a response to the debate during the first phase of the COVID-19 pandemic in Sweden, where problem gambling was suggested to be one of the health hazards potentially associated with the societal changes of the pandemic. This legislation was decided by the Swedish parliament after a proposal made by the government, and it included a maximum limit of weekly deposits made to each single operator (at a level of 5000 SEK [around 560 USD] per week) for the following 2 gambling types: (1) online casino services, which are commercialized and offered by a large number of licensed operators within Sweden, and (2) land-based electronic gambling machines, which all belong to a monopoly of a Swedish state-owned gambling operator. An additional feature of the special COVID-19–related legislation was the introduction of a 100 SEK (around 9 USD) limit to the bonuses offered to first-time clients of a gambling operator, which is typically offered by commercial online casino and sports betting services in the country [14], although not by the state-owned operator at the time of this study.

The effect of government policies on problem gambling during the COVID-19 pandemic is largely unknown. A survey study in the general population in Sweden (ie, both gamblers and nongamblers among web panel respondents) examined awareness of the COVID-19–related gambling regulation and the subjective effects of the regulation. A minority of respondents (30%) were aware of the legislation, which was however markedly more well-known among people with at least moderate-risk gambling (56% were aware of the legislation) and in the subgroup of individuals who had ever self-excluded from gambling (78% were aware of the legislation). A very low proportion of respondents reported being influenced by the regulation, with the group split between those reporting an increase or a decrease in their gambling [9]. No study has been able to examine the effects of the legislation in the specific subgroup targeted, that is, people who reach the weekly deposit limit of the legislation. In addition, it is unknown to which extent at-risk gamblers perceive such a limiting regulation as acceptable. For example, it has been suggested that deposit-limiting interventions in high-risk gambling may potentially be perceived as annoying and potentially even stimulate migration to other gambling types with a higher degree of severity [15]. Thus, there is reason to address targeted gamblers’ attitudes to this type of COVID-19–specific limit setting and determine whether such attitudes are associated with an increase or decrease in gambling.

This study was carried out by the communication and sustainability division of the state-owned Swedish gambling operator AB Svenska Spel, as a web survey targeting the group affected by the legally imposed limit on deposits. The aim of the study was to assess, in an anonymous sample of online gamblers, who reached the 5000 SEK deposit limit at the Swedish state-owned gambling operator; determine the self-reported effect of the intervention on subsequent gambling behaviors; and evaluate the knowledge about, attitudes to, and experiences of the effects of this intervention.

## Methods

### Setting

The Swedish gambling market is based on a license system, where gambling operators involved in a number of gambling types can receive a license to operate within Sweden. Operators need to follow Swedish responsible gambling legislation, including an 18-year minimum gambling age and adherence to a national government-based self-exclusion service. The self-exclusion service, administered by the Swedish Gambling Authority, allows for any individual to self-exclude from all licensed gambling (ie, with the exception of physical lotteries, including charity-based lotteries, minor gambling in funfairs, and limit-deposit “restaurant casinos” offering table games in restaurants, which constitute a very minor proportion of the Swedish gambling market). This service, described in previous publications, is a rare example of a nationwide self-exclusion service [16,17]. In Sweden, individuals with online gambling practices constitute the overwhelming majority of patients who seek treatment for a gambling disorder [18]. The prevalence of moderate-risk or problem gambling in the country has been estimated to be around 1.5%, and among these individuals, around one-third may likely meet the criteria of a disorder [19].

The state-owned operator AB Svenska Spel was traditionally a gambling monopoly and was effective in that role as long as gambling was primarily land-based. Since January 2019, the license-based system allowed for a large number of licensed operators on the Swedish market. AB Svenska Spel has one subdivision that operates in the areas of sports betting, online poker gambling, and online casino gambling, in competition with a large number of commercial operators in these areas. Another subdivision of AB Svenska Spel is a state monopoly responsible for land-based electronic gambling machines and for land-based casinos. The land-based casinos (consisting of 3 state-owned casinos in the 3 major cities of Sweden) were temporarily closed due to the COVID-19 situation from April 2020 to July 2021.

### Study Procedure

This is a self-report, electronic, anonymous survey study carried out by AB Svenska Spel. A web link was sent by email to a sample of clients who reached a 5000 SEK gambling limit (at online casino, online bingo, online sports betting, and online poker) after the introduction of the COVID-19–related gambling legislation. The survey was introduced as being sent from the gambling operator AB Svenska Spel to customers who reached this 5000 SEK deposit limit. Invited subjects were informed that the survey was confidential and that its aim was for Svenska Spel to learn more about how to improve its work against problem gambling. An incentive (a 100 SEK gift card of a type that cannot be used for gambling, alcohol, or tobacco products) was offered to respondents.

Individuals who reported being unaware of having been subject to the deposit limit were excluded from the remaining survey. The study was anonymous. Clients reaching the 5000 SEK limit were identified in the client database, where the operator has information about each client’s gambling data, but the data collection was carried out by an external consultant (the market survey company Norstat). Thus, the operator and authors were unaware which individuals responded to the survey. Age data were reported on a group level, and given the confidentiality measures, information about gender or geographical location was not collected.

As the study does not involve data that can be directly or indirectly referred to identified individuals, the project does not require ethical permission according to the Swedish ethics in research legislation. Parts of mainly descriptive data from the survey have previously been posted online in Swedish on the operator’s home page.

### Study Participants

Individuals were selected based on their gambling statistics during weeks 27 to 40 (July 2 to October 4, 2020). Individuals were addressed if they (1) had received at least one automated notification from Svenska Spel because of having deposited 5000 SEK or more during the same week in their joint gambling account involving either online casino, online bingo, online sports betting, or online poker, after the introduction of the COVID-19–related gambling legislation and (2) had lost at least 1000 SEK on online casino, online bingo, or online poker during that week. The latter was specified in order to exclude sports-only bettors who were not affected by the legislation but who may have reached the 5000 SEK limit on their joint sports and casino account of Svenska Spel. This approach identified 4782 individuals, and among them, we selected those who had not actively refused to receive client surveys and who had an email address available. This resulted in a total of 3442 potential participants. According to the funding of the study, recruitment was aimed to stop after reaching around 600 collected responses, and when recruitment was finally halted, a total of 619 responses had been received. The mean age of the participants was 45.0 years (SD 12.3 years), with a median of 45 years (IQR 35-54 years).

### Study Variables

The following study variables were collected in the study:

1. Dichotomous question about awareness of the COVID-19 legislation imposing the 5000 SEK deposit limit.
2. Likert-scale response questions about subjective self-reported effects on one’s gambling, and opinions about such an intervention and about subjective changes in a number of gambling types following the 5000 SEK limit for online casino gambling and electronic machine gambling.
3. Likert-scale response questions about the acceptability of the intervention; whether the gamblers perceived the government’s decision to introduce this intervention as “very bad,” “fairly bad,” “neither good nor bad,” “fairly good,” “very good,” or “don’t know.” These responses were dichotomized into “fairly good” or “very good” vs others and “fairly bad” or “very bad” vs others in the statistical analyses.
4. Multiple-choice questions about whether the gambler had gambled more or less on any of the following gambling types: sports betting, horse race betting, land-based restaurant casino, online poker gambling, lotteries/number games, land-based machine gambling with Svenska Spel, land-based machine gambling with other operators, and online casino with other operators; whether no other gambling type involved gambling more/less; or whether the gambler did not know.
5. Dichotomous question about whether one had gambled more or less (or did not know) after the deposit limit at Svenska Spel Sports & Casino, Svenska Spel in general, or other operators.
6. The Problem Gambling Severity Index (PGSI) [20], a validated 9-item survey tool measuring the level of risky and problematic gambling practices. The same assessment instrument has been used in general population surveys on gambling practices in Sweden [19], as well as in the COVID-19–related surveys in Sweden [8,9,21].
7. Age (in years, here reported only on a group level).

### Statistical Methods

Data were reported descriptively, and comparisons were made using the chi-square test for categorical data and Student *t* test for continuous data (and Fisher’s exact test for cross-tabulation comparisons where one or more of the squares contained less than five individuals). Finally, for the measure of whether gambling after the intervention had increased or decreased, these 2 measures were studied as outcome measures in 2 separate logistic regression analyses, where age, attitudes toward the intervention, and moderate-risk/problem gambling status were included as independent variables. Here, results were reported using odds ratios with 95% CIs. By default, associations were considered statistically significant at *P* values below .05.

## Results

### Levels of Hazardous and Problem Gambling

Full PGSI data were available from a total of 467 individuals, and of these, 25.1% (117/467) were no-risk gamblers, 20.3% (95/467) were low-risk gamblers, 31.3% (146/467) were moderate-risk gamblers, and 23.3% (109/467) were problem gamblers. In addition, another 79 individuals with partly missing answers could be identified as being at least moderate-risk gamblers, based on a PGSI score of 3 or above from available items, resulting in adequate PGSI data from 546 individuals. Thus, in the full sample of 619 individuals, 54% (n=334) were at least moderate-risk gamblers and 34% (n=212) were no-risk or low-risk gamblers, with missing data in the remaining 73 individuals for whom the total score from available PGSI items was 0 to 2. Moderate-risk/problem gamblers were significantly younger than others (43.5 vs 48.3 years, *P*<.001).

### Awareness and Acceptability of the Government Regulation

Overall, 60.1% (372/619) were aware of the limit for their deposits by the government’s COVID-19–specific gambling regulation. Awareness of the regulation was higher among individuals with moderate-risk/problem gambling than among others (68% vs 48%; *P*<.001), but was unrelated to age (44.5 vs 45.6 years; *P*=.29). In the entire sample, 34.6% (214/619) believed that the regulation was very good, 19.2% (119/619) believed that it was fairly good, and 21.0% (130/619) believed that it was neither good nor bad. Moreover, 9.5% (59/619) stated that it was fairly bad and 13.9% (86/619) stated that it was very bad. Furthermore, 1.8% (11/619) reported that they did not know.

### Gambling After the COVID-19 Regulation in Individuals With Awareness of the Regulation

After reaching the 5000 SEK limit, among those reporting being aware of the regulation (n=372), 38.7% (144/372) stated that this had decreased their total gambling, 47.3% (176/372) stated that their gambling remained approximately the same as before, 7.8% (29/372) believed it increased their gambling, and 6.2% (23/372) did not know.

Gambling types perceived to have increased (in other operators) after the intervention were horse race betting (29/372, 7.8%), sports betting (52/372, 14.0%), lotteries and “number games” (18/372, 4.8%), online casino (123/372, 33.1%), online bingo (12/372, 3.2%), online poker (12/372, 3.2%), “restaurant casinos” (7/372, 1.9%), land-based electronic gambling machines of Svenska Spel (29/372, 7.8%), and other land-based electronic gambling machines (13/372, 3.5%). Additionally, 37.4% (139/372) did not perceive themselves to have increased any gambling type and 9.7% (36/372) did not know.

Moreover, 37.6% (140/372) reported that they decreased their gambling on the Svenska Spel Sports & Casino subdivision, 19.4% (72/372) reported that they decreased their gambling on Svenska Spel overall, 19.4% (72/372) reported that they decreased their gambling at other operators, and 30.6% (114/372) did not know.

We found that 13.4% (50/372) had gambled on casino, poker, or bingo games at only 1 operator since July 1 (since the introduction of the intervention), 55.6% (207/372) had gambled on these games at 2 to 4 operators, 21.2% (79/372) had gambled at 5 to 10 operators, 3.0% (11/372) had gambled at 11 to 15 operators, 2.7% (10/372) had gambled at more than 15 operators, and 4.0% (15/372) did not know. Thus, altogether, 83% had gambled on casino, poker, or bingo games on more than one gambling operator after the deposit limit. Moreover, 40.6% (151/372) reported being limited, following the COVID-19 regulation, from another operator than Svenska Spel during the observation period, 53.2% (198/372) reported not being limited, and 6.2% (23/372) did not know.

### Comparison of Moderate-Risk/Problem Gamblers and Others

Among individuals reporting being aware of the COVID-19 regulation, 284 had full data for the PGSI and 327 had full data in the dichotomy of moderate-risk/problem gambling vs no-risk/low-risk gambling. The number of gambling operators reported was strongly associated with moderate-risk/problem gambling (*P*<.001, chi-square linear-by-linear). Among those reporting two or more other gambling operators after reaching the weekly limit at Svenska Spel, moderate-risk/problem gambling was detected in 74% compared with 39% among those reporting 1 operator (*P*<.001).

Differences between moderate-risk/problem gamblers and other gamblers are demonstrated in Table 1. Altogether, moderate-risk/problem gamblers were significantly more likely to report an increase (following the intervention) in Svenska Spel machine gambling, online casino at other operators, and lotteries and number games, and they were markedly less likely to report that they had not increased gambling at other operators, whereas no differences were seen for other gambling types. Moderate-risk/problem gambling was not significantly associated with having decreased Svenska Spel gambling or Svenska Spel Sports & Casino gambling.

Altogether, moderate-risk/problem gamblers were significantly more likely than others to report having increased overall gambling after the introduction of the regulation, and were not more likely to report overall decreased gambling due to the present regulation. They were significantly more likely to perceive the regulation as very good or fairly good, and less likely to perceive it as very bad or fairly bad.

**Table 1:** Statistical comparisons between moderate-risk/problem gamblers and other gamblers (N=327).

Variable			Moderate-risk/problem gamblers (n=226), n (%)		Other gamblers (n=101), n (%)		*P* value
**Postintervention increase**							
	Svenska Spel machine gambling	22 (9.7)		3 (3.0)		.04	
	Online casinos, other operators	95 (42.0)		19 (18.8)		<.001	
	Lotteries and number games	15 (6.6)		0 (0.0)		<.007	
	Non-Svenska Spel land-based machine gambling	7 (3.1)		4 (4.0)		.74	
	Restaurant casino gambling	4 (1.8)		1 (1.0)		>.99	
	Online poker, other operators	8 (3.5)		2 (2.0)		.73	
	Online bingo, other operators	8 (3.5)		1 (1.0)		.28	
	Sports betting	36 (15.9)		10 (9.9)		.15	
	Horse race betting	17 (7.5)		10 (9.9)		.47	
Postintervention increase in gambling overall			24 (10.6)		2 (2.0)		.007
No postintervention increase in gambling on other operators			66 (29.2)		59 (58.4)		<.001
**Postintervention decrease**							
	Svenska Spel gambling overall	47 (20.8)		19 (18.8)		.68	
	Svenska Spel Sports & Casino gambling overall	85 (37.6)		39 (38.6)		.86	
Postintervention decrease in gambling overall			88 (38.9)		37 (36.6)		.69
Perceived intervention as very good or fairly good			126 (55.8)		42 (41.6)		.02
Perceived intervention as very bad or fairly bad			53 (23.5)		40 (39.6)		.003

### Correlates of Reporting Increased or Decreased Gambling Following the Intervention

Age was unrelated to the reporting of decreased gambling after the regulation (45.9 vs 43.7 years, *P*=.09), but the group reporting increased gambling after the regulation was significantly younger (38.1 vs 45.1 years, *P*<.01). Age was unrelated to the perception of the regulation as very good/fairly good (*P*=.86) or very bad/fairly bad (*P*=.82). Age was also unrelated to a decrease in gambling on Svenska Spel Sports & Casino (*P*=.68), Svenska Spel overall (*P*=.52), and other operators (*P*=.78).

The reporting of increased or decreased gambling following the intervention was associated with attitudes toward the regulation. Among those who perceived the regulation as very good or fairly good, 53.1% (102/192) reported decreased gambling (compared to 23.3% [42/180] among others; *P*<.001) and 4.7% (9/192) reported increased gambling (compared to 11.1% [20/180] among others; *P*=.02). Among those who perceived the regulation as very bad or fairly bad, 18.1% (19/105) reported decreased gambling (compared to 46.8% [125/267] among others; *P*<.001) and 15.2% (16/105) reported increased gambling (compared to 4.9% [13/267] among others; *P*<.001).

In logistic regression analysis (Table 2), a reported increase in gambling following the intervention (n=26) was significantly associated with the opinion that the regulation was very bad or fairly bad and with being a moderate-risk/problem gambler, and was nearly significantly associated with younger age. A reported decrease in gambling following the intervention (n=125) was associated with the opinion that the regulation was very good or fairly good, but not with being a moderate-risk/problem gambler or with age (Table 2).

**Table 2:** Logistic regression of reported increased gambling or decreased gambling following the intervention (N=327).

Variable		OR^a^ (95% CI)		*P* value
**Associations with the reporting of increased gambling following the intervention (n=26)**				
	Regulation perceived as very bad or fairly bad	4.39 (1.88-10.30)	<.001	
	Moderate-risk/problem gambler	6.89 (1.53-31.00)	.01	
	Age	0.96 (0.93-1.00)	.05	
**Associations with the reporting of decreased gambling following the intervention (n=125)**				
	Regulation perceived as very good or fairly good	3.43 (2.12-5.54)	<.001	
	Moderate-risk/problem gambler	1.02 (0.60-1.73)	.94	
	Age	1.02 (1.00-1.04)	.10	

^a^OR: odds ratio.

## Discussion

### Principal Findings

This study examined self-report of gambling among individuals after online gambling was limited due to a special COVID-19–related gambling regulation imposing a weekly deposit limit on online casino and electronic gambling. The study demonstrated that acceptability of the intervention was generally fair. Although only 6 out of every 10 gamblers reported being aware of the limit introduced by the regulation, a majority perceived the intervention as generally positive. In those who were aware of this, it was considerably more common to perceive that the intervention had decreased their overall gambling than to perceive that it increased their gambling. However, limitations and challenges of the intervention were demonstrated. A large majority gambled at other operators, where 4 out of every 10 gamblers reached the regulated weekly deposit limit. This challenge was more pronounced for online casino, which was by far the gambling type most commonly reported to have increased at other gambling sites after being limited by the present operator. This sample of online gamblers who reached the weekly limit had very high rates of gambling problems, and those who scored positive for at least moderate-risk gambling were considerably more common to report online casino gambling at other sites after being limited, to gamble at more operators, and to perceive themselves to have increased gambling after the introduction of the weekly limit. A self-reported negative effect from the intervention was associated with negative attitudes toward it and with higher gambling problems.

Awareness of the fact that one had reached the deposit limit imposed by the government regulation may seem surprisingly low; only 6 out of every 10 gamblers reported that they were aware. However, consistent with previous general population data assessing knowledge about government policies related to gambling and COVID-19 [9], this awareness was markedly higher in the subgroup with manifest gambling problems. Thereby, it can be assumed that people with highly hazardous gambling patterns constitute a group with particularly high gambling involvement and therefore a higher likelihood of recognizing and understanding the nature of the present intervention. Likewise, it can be argued that the intervention imposed by the government aims particularly at this group, although it also indicates that awareness, and therefore efficacy, of the intervention may have the potential to increase in gamblers without detected gambling problems.

A subgroup of respondents here reported an increase in gambling subsequent to the intervention (ie, intuitively a negative and unintended effect of the intervention). The reporting of an overall increase in gambling as a consequence of the intervention was rare, but was several times more common in moderate-risk or problem gamblers than in others. Thus, although only a minority reported a markedly deteriorating effect from this intervention, it is clear that individuals with a likely addictive behavior were at higher risk of having experienced such a negative effect in contrast to the intentions of the intervention. It cannot be concluded that an actual increase in gambling was indeed a consequence of the temporary COVID-19 legislation, but again, individuals with higher levels of gambling problems may be at a particular risk of having an unfavorable course in gambling as a reaction to the crisis. In previous research involving prevention or harm reduction methods in problem gambling, it had been stated that subgroups of gamblers may perceive preventive interventions as annoying in a way that would, theoretically, even worsen their gambling practices. While this has been described as unlikely, it has been stated that problem gamblers may be at higher risk of such reactions than other gamblers [15]. Given the very high levels of gambling and detected gambling problems in this study, it cannot be excluded that such negative reactions to an intervention are responsible for one part of the perceived negative effect in some gamblers. Here, it should be kept in mind that a significant proportion of the study sample expressed negative feelings related to this type of intervention, but somewhat contrary to what could be expected, moderate-risk/problem gamblers had somewhat more favorable attitudes toward it.

The present findings may be considered to corroborate previous findings that individuals who report an increase in gambling during these times of COVID-19 have a markedly higher probability of being problem gamblers [8,21]. While the gambling increase was not measured in relation to the pandemic itself, but in relation to a specific regulation in affected gamblers, it further demonstrates that people with hazardous gambling practices are more likely than others to demonstrate a negative development during the COVID-19 crisis. Likewise, in a survey study among gamblers in the population, those who gambled even in highly restricted gambling types during the lockdown period when sports were generally cancelled, were a group with markedly higher gambling problems [21]. Among US casino gamblers, when casinos closed, a minority migrated to new types of gambling, but this group demonstrated more problematic levels of gambling [22]. Thus, as a general rule from these studies conducted during COVID-19, including the present setting, high levels of gambling during these times of crisis are more likely to be associated with problematic gambling behaviors.

Meanwhile, it should be kept in mind that all studies in the area have not demonstrated an increasing trend in gambling during the pandemic. In Australia, for example, an increase was not seen [23], and in a Canadian study of land-based casino gamblers, during casino closure, a migration toward online gambling was seen primarily among those who had engaged in online casino gambling before, rather than in the full population [24]. Likewise, early observations from the very first phase of the pandemic did not display obvious increases in online gambling activity [5,6]. In line with this, subgroups of gamblers with manifest gambling problems have even reported the COVID-19 period as relieving, due to a decrease in gambling opportunities during some phases [25]. Thus, it is unclear whether the increase in gambling in a subgroup of respondents here and in response to the imposed deposit limit, may be due to pandemic-related effects or the intervention itself, or simply because of a generally increasing course in the gambling practices of these specific individuals.

It can be debated whether the present data support an effect from the type of intervention imposed or whether the nature of the intervention only may invite gamblers to migrate to other gambling types. Again, it is clear from the present data that at least for some gambling types, individuals with risky gambling habits are more likely than others to transfer their gambling to other modalities or gambling types. This corroborates the findings of survey studies in the population, where individuals who increase another gambling type in response to a limit to one gambling type (in this case, the limitation of sports betting during the lockdown period) are more likely to have gambling problems [8,21]. The present type of intervention appears to have at least a relatively high level of acceptability in affected individuals. A majority had a favorable attitude toward it, and a minority claimed to be against it. However, although acceptability was relatively high, only around 1 in 5 respondents believed they had decreased their gambling with other operators due to the intervention. Thus, while the intervention does not technically prevent an individual from continuing to a different gambling operator after reaching the COVID-19–related limit at the first one, the signaling value of the intervention might decrease gambling at other gambling sites. Here, it should again be remembered that the study sample generally involved a very high level of gambling problems, and it can be suspected that the enforcement of a deposit limit may not necessarily invite reflection and a motivational process of change in the individual in the short term.

Three gambling types stood out as being more commonly reported in problem gamblers owing to an increase in gambling practiced in response to the imposed deposit limit. This included machine gambling and lotteries/number games, as well as online casinos at other operators. Online casino was the most commonly cited. The large role of online casinos in problem gamblers in the present setting has been documented previously; for example, it is by far the most common gambling type reported in clients seeking treatment at a regional gambling unit in Sweden [18]. Thus, it is of great interest to conclude that when gamblers are temporarily banned from one online operator service due to the weekly deposit limit, they most commonly turn to this gambling type but at other operator sites. Moreover, again, it confirms the addictive potential of online casino gambling, which is a highly accessible, rapid, and repetitive type of gambling, as the proportion reporting an increase in that gambling type at other operators was markedly more common among individuals with gambling problems.

Our study sample had very high rates of gambling problems. More than half of the full sample represented at least moderate-risk gamblers, and for the further items studied in the subgroup with awareness of the intervention, moderate-risk gamblers made up a large majority. This is further supported by the fact that a majority reported gambling at more operators, even to the extent that 2 of every 5 respondents had experience of reaching the imposed limit at a different operator. It can be argued that the intervention therefore specifically addressed the targeted group, and therefore, from this study, less is known about whether the intervention plays any role in the remaining population (ie, among people with low nonhazardous gambling practices). Over and above the actual effect of the intervention in those facing the limit, it can be argued that an intervention of this nature may have a didactic effect in individuals without current gambling problems, but who may potentially benefit from advice or from the political signal that gambling is a product with addictive potential and has the risk of severe harm during COVID-19.

### Limitations

Owing to the confidentiality protocol applied in this survey study, more detailed data, such as gender, geographical location, and previous gambling habits, were not collected. Thus, individual responses could not be linked to any identifying information or to any prior gambling statistics in the databases of Svenska Spel, for whom the identity of respondents remained unknown. While this successfully maintained confidentiality of the respondents, more in-depth data on risk factors could not be detected, and response data clearly rely on the self-report of participants. As in all self-report surveys, the risk of recall bias or other misinformation cannot be disregarded. Moreover, the limited number of participants, which included the first individuals who responded to the invitation to participate, may constitute a risk of bias, as individuals responding first may potentially have a different degree of involvement in these issues and therefore potentially have a different gambling pattern or different opinions than others. Moreover, it should be kept in mind that the population assessed here was recruited from a single gambling operator, and although it operates in diverse areas, such as sports betting, poker gambling, and chance-based rapid online games (eg, casino slots and bingo), its profile as a state-owned gambling operator may potentially attract a somewhat different group of gamblers than certain other operators in the market.

A strength of the study is that it, quite uniquely, had the ability of addressing one operator’s clients with respect to their gambling at other operators, a type of data that cannot easily be obtained from other sources. The present group is also less likely to be examined in detail in larger population surveys, where the group makes up a small minority with extreme gambling patterns, but it could be assessed here. Moreover, the study had the advantage of being able to address a new COVID-19–specific intervention in relatively temporal proximity to the introduction of the intervention.

### Conclusions

In a high-level gambling sample exposed to a government-imposed weekly deposit limit aiming to prevent potential COVID-19–related gambling issues, the acceptability of the intervention was relatively high and somewhat higher in problem gamblers, although many exposed individuals were not apparently aware of having been subject to the intervention. The challenges of a single-operator weekly deposit limit were obvious; many of these exposed individuals subsequently gambled at other operators, and in many cases, they gambled at many operators. Self-reported improvement from the intervention was common and self-reported negative effects were rare, but risk gamblers demonstrated a much higher rate of negative effects and, in particular, a high prevalence of online casino gambling at other operators after being limited by the imposed intervention at the operator studied here. Promising attitudes toward this kind of deposit-limit intervention were seen, but the study identified some difficulties, and it may inspire future development of further types of interventions addressing the overall problem of high-risk gamblers. High-level online gamblers constitute a group with great needs, and the gambling operator’s own monitoring may identify this group with potential for receiving harm-reducing and therapeutic interventions.

## Abbreviations

PGSI: Problem Gambling Severity Index
